# Single-cell RNA-seq data analysis using graph autoencoders and graph attention networks

**DOI:** 10.3389/fgene.2022.1003711

**Published:** 2022-12-09

**Authors:** Xiang Feng, Fang Fang, Haixia Long, Rao Zeng, Yuhua Yao

**Affiliations:** ^1^ College of Information Science Technology, Hainan Normal University, Haikou, Hainan, China; ^2^ College of Information Engineering, Hainan Vocational University of Science and Technology, Haikou, Hainan, China; ^3^ College of Mathematics and Statistics, Hainan Normal University, Haikou, Hainan, China

**Keywords:** single-cell RNA-seq, gene imputation, cell clustering, graph neural networks, graph autoencoders, graph attention networks

## Abstract

With the development of high-throughput sequencing technology, the scale of single-cell RNA sequencing (scRNA-seq) data has surged. Its data are typically high-dimensional, with high dropout noise and high sparsity. Therefore, gene imputation and cell clustering analysis of scRNA-seq data is increasingly important. Statistical or traditional machine learning methods are inefficient, and improved accuracy is needed. The methods based on deep learning cannot directly process non-Euclidean spatial data, such as cell diagrams. In this study, we developed scGAEGAT, a multi-modal model with graph autoencoders and graph attention networks for scRNA-seq analysis based on graph neural networks. Cosine similarity, median L1 distance, and root-mean-squared error were used to measure the gene imputation performance of different methods for comparison with scGAEGAT. Furthermore, adjusted mutual information, normalized mutual information, completeness score, and Silhouette coefficient score were used to measure the cell clustering performance of different methods for comparison with scGAEGAT. Experimental results demonstrated promising performance of the scGAEGAT model in gene imputation and cell clustering prediction on four scRNA-seq data sets with gold-standard cell labels.

## Introduction

In recent years, use of single-cell RNA sequencing technology (scRNA-seq) in research has become increasingly popular. scRNA-seq can be used to assess gene expression of cells at the single-cell level and sequence genes from uncommon or rare cells. The sequencing information allows identification of unknown cell types based on specific gene expression, which is a significant advantage in brain cell differentiation and embryonic cell development research. In addition, scRNA-seq data plays an important role in guiding the diagnosis and treatment of disease (Potter, 2018). With the development of high-throughput sequencing technology, the scale of scRNA-seq data has surged; it is typically high-dimensional, high noise, high sparsity data. Therefore, analyzing scRNA-seq data has become an important research direction in bioinformatics (Abdelaal et al., 2019).

A remarkable feature of scRNA-seq data is its many zero values, resulting in high data sparsity. Some of the zero values in scRNA-seq data reflect a true lack of gene expression, but other zero value genes are mistakenly quantified as not expressed due to transcriptome deletion or low expression, which results in a large number of zero values, or dropout events (Risso et al., 2018; Xu et al., 2020a). In recent years, many methods have emerged for imputation of scRNA-seq data, such as Magic (Van Dijk et al., 2018), Saver (Huang et al., 2018), scImpute (Li and Li, 2018), and others. These methods correct the inaccurate zero read count by borrowing the information from similar genes or cells and restoring the gene expression data in the scRNA-seq output. Magic uses similar cells and gene information to impute missing values based on the Markov transfer matrix (Van Dijk et al., 2018).

In contrast, Saver uses the relationship between genes to infer the gene expression after denoising by using the Bayesian method (Huang et al., 2018). Magic and Saver can restore the expression level of each gene in each cell, including non-zero values. The scImpute method computes the dropout probability of each gene in each cell by fitting the mixed model and then estimates the dropout value in cells by comparing with information on the same gene obtained from similar cells (Li and Li, 2018). However, Magic and Saver have failed to learn the nonlinear relationship and counting structure in scRNA-seq data. With the development of deep learning, imputation methods-based neural networks have been proposed, including a deep count autoencoder (DCA) (Eraslan et al., 2019), DeepImpute (Arisdakessian et al., 2019), and Saver-X (Wang et al., 2019). DCA proposes a deep count autoencoder for single-cell RNA-seq denoising, which uses a negative binomial noise model with or without zero-inflation to account for the count distribution, overdispersion, and sparsity of the results, and allows nonlinear gene-gene dependencies to be captured (Eraslan et al., 2019). DeepImpute uses the divide-and-conquer approach to impute groups of target genes using other genes that are strongly associated with the target genes (Arisdakessian et al., 2019). Saver-X extracts the relationship between transferable genes by combining deep autoencoders with a Bayesian model to impute the scRNA-seq data set (Wang et al., 2019). However, these methods cannot directly deal with non-Euclidean spatial data, such as cell maps.

An important phase in the analysis of scRNA-seq data is cell clustering. Cell clustering classifies cells into different groups according to the cell-cell distance matrix, so cells of highest similarity can be clustered into groups sorted to the greatest extent possible. Clustering aims to explore or identify cell types or sub-types and reveal complex structure and potential functions of various tissues (Peng et al., 2020). Cell clustering is also the premise of scRNA-seq downstream analysis. Some classical clustering methods, such as spectral clustering (Ntranos et al., 2016), double clustering (Shi and Huang, 2017), sparse subspace clustering (Zhuang et al., 2021), and some other hybrid clustering models (Sun et al., 2018), show good clustering performance when dealing with small-scale single-cell data sets. However, with the improvement of sequencing technology, the scale of single-cell sequencing data has expanded in recent years. These classical clustering methods are inefficient in analysis of large-scale data, and many clustering methods cannot accurately manage complex single-cell data. Many scholars have turned to the design of supervised and unsupervised clustering techniques based on deep learning for scRNA-seq. Li et al. (2020)proposed a deep embedding algorithm based on autoencoders for clustering scRNA-seq data with a self-training target distribution that can also denoise and potentially remove batch effects. Chen et al. (2020)presented an end-to-end supervised clustering and cell annotation framework, scAnCluster, built upon their previous unsupervised clustering work. Tian et al. (2019) created the scDeepCluster method, which uses a nonlinear approach to combine DCA modeling and the Deep Embedding algorithm for Single-Cell Clustering (DESC). The method seeks to improve clustering performance while reducing dimensions directly. The scDeepCluster outperforms state-of-the-art approaches on a variety of clustering efficiency metrics. Grønbech et al. (2020) introduced a single variational autoencoder (scVAE) model, which is used to cluster cells. Since scVAE uses raw count data as input, many traditional preprocessing steps are not required. The scVAE can accurately predict each cell’s expected and implicit gene expression and is flexible in use of the known scRNA-seq count distribution (such as the Poisson distribution or Negative Binomial distribution) as its model hypothesis.

This article introduces a graph neural networks framework based on multi-modal graph autoencoders (GAEs) and graph attention (GAT) networks (scGAEGAT) to model heterogeneous cell-cell relationships and their underlying complex gene expression patterns from scRNA-seq data. The scGAEGAT model performs gene imputation and cell clustering, uses an encoder-decoder deep learning framework for scRNA-seq analysis, and provides a global perspective for exploring cell relationships by capturing the associations between cells across the entire cell population.

## Materials and methods

### scGAEGAT model

**FIGURE 1:**
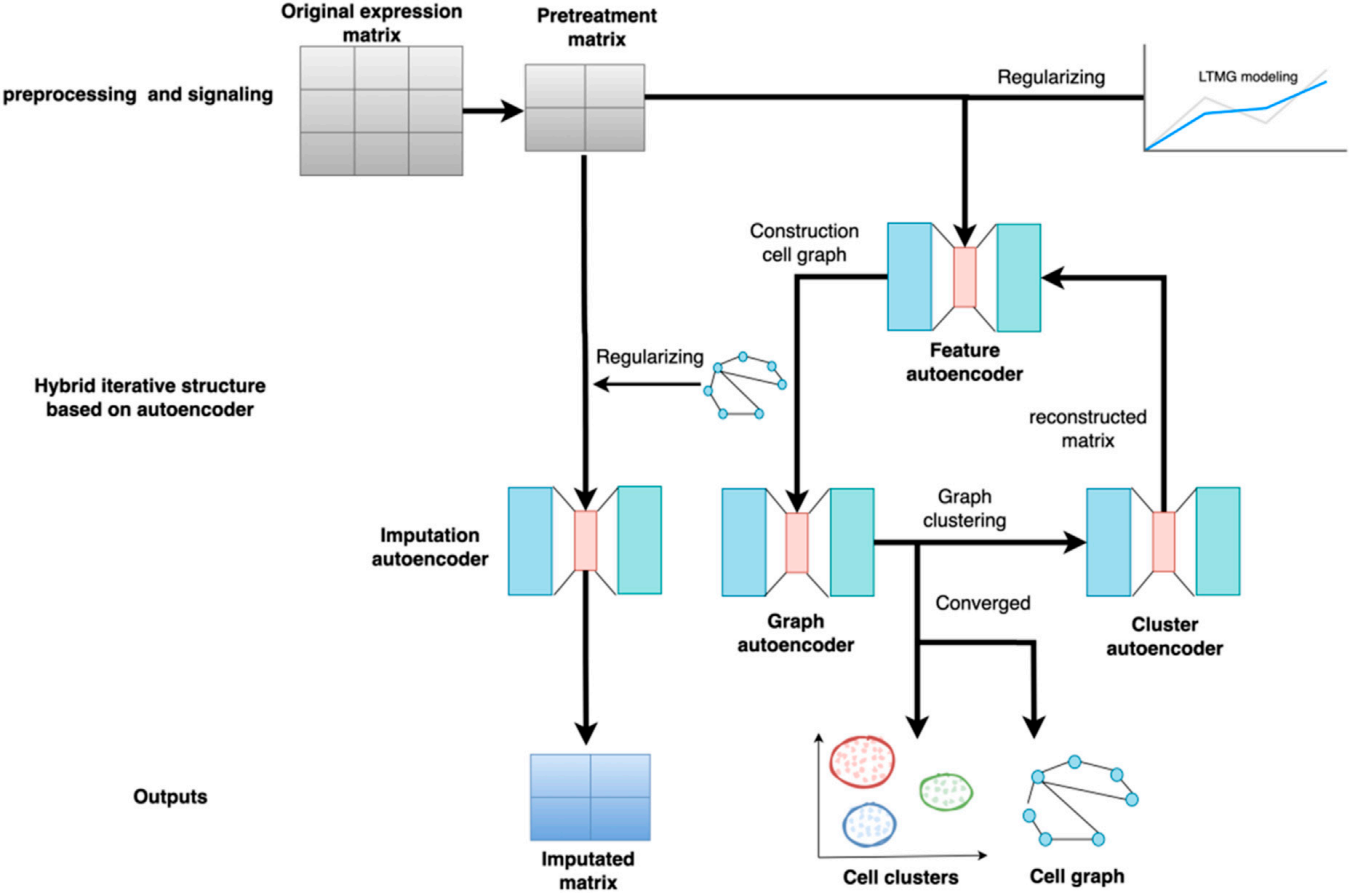
Structure of the scGAEGAT model. The model uses the pretreatment gene expression matrix as input to the feature autoencoder. The feature autoencoder builds and prunes the cell graph through learned embedding. The graph autoencoder adds the graph attention mechanism. It takes the constructed cell graph as input by adding different weights to different nodes, which can better capture cell relationships and achieve cell-type clustering. Each type of cell has a separate cluster autoencoder to reconstruct the gene expression value of the cell. The reconstructed gene expression value is used as the new input for iteration until convergence.

The overall structure of the scGAEGAT model is shown in Figure 1. scGAEGAT is a graph neural network that integrates GAEs and GAT networks. Compared with the Magic (Van Dijk et al., 2018) and DCA (Eraslan et al., 2019) methods, scGAEGAT contains the GAT mechanism, which can allocate different weights to different cells through the coefficient of GAT. In the iteration process, scGAEGAT comprises four powerful autoencoders: a feature autoencoder for gene expression regulation, a graph autoencoder for constructing cell-cell relationships, a cluster autoencoder for identifying cell type, and an imputation autoencoder for recovery of the gene expression matrix. scGAEGAT obtains the topological information between genes to express the cell-cell relationship through low dimensional embedding of multiple autoencoder structure training feature vectors. It plays a significant role in aggregating cell types, inferring cell arrangement according to trajectory topology, and imputing missing information to improve the correlation between genes.

**FIGURE 2:**
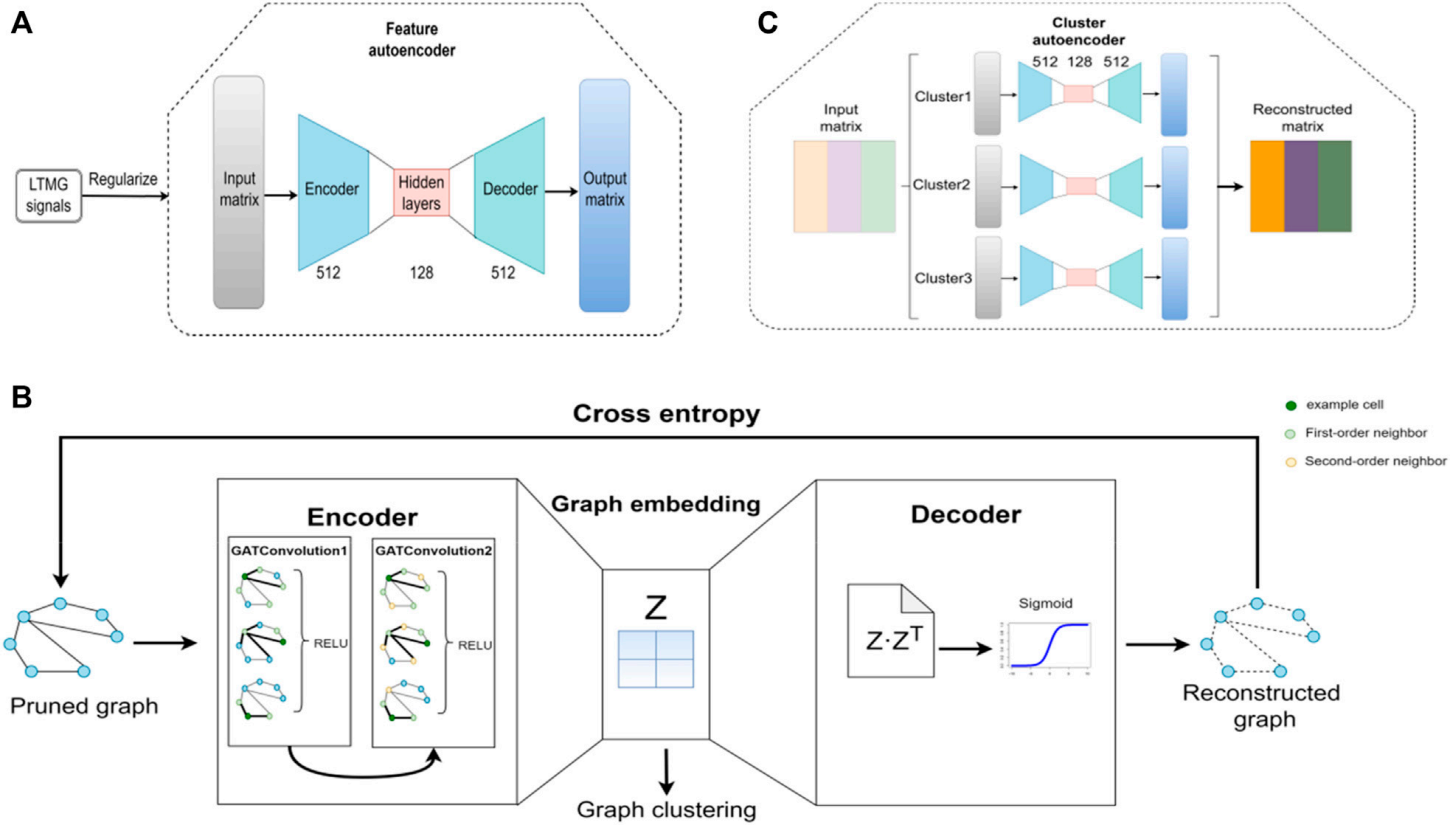
Architecture of the scGAEGAT model. **(A)** Structure of the feature autoencoder. **(B)** Structure of the graph autoencoder. **(C)** Structure of the cluster autoencoder.

The structure of the feature autoencoder is shown in Figure 2A; it takes the gene expression matrix, composed of the first 2000 genes obtained after removing the low-expression cells and genes, and sorts them according to the standard deviation as input. The feature autoencoder regulates the regulatory signals between genes through the left-truncated mixed Gaussian (LTMG) model. The purpose of using the LTMG model (Wan et al., 2019; Xie et al., 2020) as a regularizer is to carry out processing according to the regulation state of each gene through the loss function. The encoder size of the feature autoencoder is 512 × 128, and the size of the decoder is 128 × 512. By reducing the loss function, the reconstructed gene expression matrix is as similar as possible to the raw gene expression matrix to achieve the training feature of the autoencoder. The K-Nearest Neighbor (KNN) graph constructs a cell graph through the learned embedding, in which nodes represent cells and edges represent the relationship between cells (Bendall et al., 2014; Wolf et al., 2019). Then, by removing the noisy edges in the cell graph, the adaptive number of neighbor nodes is selected for the KNN graph to prune the cell graph.

The structure of the GAE is shown in Figure 2B, which takes the trimmed cell diagram as input. The encoder is composed of two GAT convolution layers. By adding a GAT mechanism, different weights are added to other nodes to learn the graph embedding of nodes. In the encoder, each node of the cell graph gathers the information of its neighbor nodes to understand the low-dimensional embedding of the pruned cell graph. The decoder regenerates the cell graph through the sigmoid activation function and continuously optimizes the model by minimizing cross-entropy loss. Then the k-means method is used to cluster cells according to the learned graph embedding and the Louvain algorithm is used to determine the number of cell clusters (Wang et al., 2021).

The structure of the cluster autoencoder is shown in Figure 2C, with the reconstructed gene expression matrix generated by the feature autoencoder as input. The expression matrix in each cell cluster is reconstructed by the cluster autoencoder. The cluster autoencoder processes different cell types according to the cell types learned by the GAE. Each cell type has a single autoencoder for separate training. There is continuous iteration through the feature autoencoder, GAE, and cluster autoencoder; when the clustering result does not change, the iteration stops, and the cell clustering result is the final cell cluster prediction result.

When the iteration stops, the imputation autoencoder takes the pretreated gene expression matrix as the input and is trained by regularization of the inferred cell graph and cell type. The regularization matrix is generated by the edges of the cell graph learned in the iteration process and *via* recognized cell types. Finally, the reconstructed gene expression value output is the final imputation result.

### Data preprocessing and normalization

The scGAEGAT model takes the gene expression matrix of scRNA-seq data as input. Because there is a high dropout rate in scRNA-seq data, it is necessary to filter and control the quality of scRNA-seq data first, then the genes are sorted according to the standard deviation, and finally, the first 2,000 genes are extracted for research. All data are normalized by log-transformation. After preprocessing, the LTMG model (Wan et al., 2019) is used to accurately infer the modality and distribution of individual gene expression profiles in scRNA-seq data, modeling the dropout and low expressions as left-censored data caused by a limited experimental resolution. The LTMG model is adopted to the top 2,000 variable genes to quantify gene regulatory signals encoded among diverse cell states in scRNA-seq data. This model was built based on the kinetic relationships between the transcriptional regulatory inputs and mRNA metabolism and abundance, which can infer the expression of multi-modalities across single cells. The captured signals have a better signal-to-noise ratio to be used as a high-order restraint to regularize the feature autoencoder. For *N* cells, the normalized expression values of gene *X* are denoted as $X=\left\{x_{1},x_{2},\cdots ,x_{N}\right\}$ , supposing $x_{j}\in X$ adhere to a mixture of k Gaussian distributions, corresponding to k possible gene transcriptional regulatory signals (TRS). The density function of gene X is:

$$\rho \left(X;\theta \right)=\overset{N}{\underset{j=1}{\prod }}\rho \left(x_{j};\theta \right)=\overset{N}{\underset{j=1}{\prod }}\overset{k}{\underset{i=1}{\sum }}\alpha_{i}\rho \left(x_{j};\theta_{i}\right)=\overset{N}{\underset{j=1}{\prod }}\overset{k}{\underset{i=1}{\sum }}\alpha_{i}\frac{1}{\sqrt{2\pi \sigma_{i}}}e^{\frac{{-\left(x_{j}-\mu_{i}\right)}^{2}}{2\sigma_{i}^{2}}}=L\left(\theta ;X\right)$$ (1)

where $\alpha_{i}$ is the mixing probability, $\mu_{i}$ is the average value, $\sigma_{i}$ is the standard deviation of the ith Gaussian distribution, N is the total number of observations, and θ can be obtained by EM algorithm of k value to model the errors at zero and the low expression values. With the left truncation assumption, the gene expression profile is split into *M*, which is a truly measured expression of values, and *N − M* represents left-censored gene expressions for *N* conditions. The parameter θ maximizes the likelihood function and can be estimated by an expectation-maximization algorithm. The number of Gaussian components is selected by the Bayesian Information Criterion; the original gene expression values are then labeled as the most likely distribution under each cell. The probability that $x_{j}$ belongs to distribution *i* is formulated by:

$$p\left(x_{j}\in TRS\;i/K,\theta *\right)\epsilon \frac{\alpha_{i}}{\sqrt{2\pi \sigma_{j}^{2}}}e^{\frac{{-\left(x_{j}-\mu_{i}\right)}^{2}}{2\sigma_{i}^{2}}}$$ (2)

where $x_{j}$ is labeled by TRS i if $p\left(x_{j}\in TRS\;iK,\theta *\right)=\max_{i=1....k}\left(p\left(x_{j}\in TRS\;iK,\theta *\right)\right)$ . Thus, the discrete values (*1,2, … ,K*) for each gene are generated.

### Feature autoencoder

The feature autoencoder is composed of an encoder and decoder process. It takes the gene expression matrix as input, and LTMG performs regularization processing to learn the embedding expression of scRNA-seq data. The encoder constructs the embedding of low dimension $\hat{X}$ (reconstructed gene expression matrix) through the input gene expression matrix X (normalized gene expression matrix), then the decoder is reconstructed according to embedding, so the encoder is a process of dimension reduction. The feature autoencoder is trained by minimizing the loss function of the difference between the input gene expression matrix and the output matrix so that the output matrix is as similar as possible to the input matrix. The mean square error (MSE) is defined as:

$$\sum {\left(X-\hat{X}\right)}^{2}$$ (3)

### Graph autoencoder

The GAE adopts a deep learning model based on GAT, which can process input as a sparse matrix. Due to the addition of a GAT convolution layer, the gathering of cell information is improved. The calculation process of graph convolutional layers is as follows:

$$H^{\left(k+1\right)}=f\left(H^{\left(k\right)},A\right)=\sigma \left(\tilde{A}H^{\left(k\right)}W^{\left(k\right)}\right)$$ (4)

where $\tilde{A}=D^{-1/2}AD^{1/2}$ , A and D are the adjacency matrix and degree matrix of the cell-cell connection graph, respectively, k is the number of layers of graph convolution, W is the learning weight, σ is the activation function, and $H^{\left(k\right)}$ is the input matrix of the convolution of the kth layer graph. The GAE is composed of an encoder and a decoder. We added two layers of graph convolution to the encoder, and the output of the encoder is:

$$H^{\left(2\right)}=ReLU\left(\tilde{A}ReLU\left(\tilde{A}\hat{X}W_{1}\right)W_{2}\right)$$ (5)

where $W_{1}$ is the learning weight of the first layer and $W_{2}$ is the learning weight of the second layer. The first and second-level output dimensions are set to 32 and 16, respectively, and the learning rate is set to 0.001. To better learn the cell information, we introduced the attention model, which adds attention to the neighbor nodes by increasing the weight, to enhance GAE learning of the embedding of the cell graph. The formula for calculation of the attention coefficient is:

$$e_{ij}=a\left(\vec{h_{i}},\vec{h_{j}}\right)=W\vec{h_{i}}∙W\vec{h_{j}}$$ (6)

where $\vec{h_{i}}$ and $\vec{h_{j}}$ represent the characteristics of node *i* and node *j*, respectively, the attention coefficient of node *i* to node *j*, and *W* is the shared weight matrix. It can change the features of nodes into higher-level features so that the pruned cell map obtains better expression ability. Adding multiple independent attention coefficients can extend further to the multi-head attention mechanism. By calculating the average value from the adaptive attention coefficient, more stable learning of the embedding and topology information of the cell graph can occur. The formula of the multi-head attention mechanism is:

$${\vec{h}}^{\prime }=\sigma \left(\frac{1}{K}\overset{K}{\underset{k=1}{\sum }}\underset{K\epsilon N_{i}}{\sum }a_{ij}^{k}W^{k}\vec{h_{i}}\right)$$ (7)

To compare the attention coefficients of different nodes, we added the softmax function for standardization. The decoder of the GAE is defined as:

$$\hat{A}=sigmoid\left(ZZ^{T}\right)$$ (8)

where $\hat{A}$ is the reconstructed adjacency matrix of *A*, and Z is the embedding learned by the encoder. The function of the graph self-encoder is to minimize the difference between the input matrix A and the output matrix $\hat{A}$ , which can be achieved by minimizing the cross entropy L :

$$L\left(A,\hat{A}\right)=-\frac{1}{N\times N}\overset{N}{\underset{i=1}{\sum }}\overset{N}{\underset{j=1}{\sum }}\left(a_{ij}*\log\left({\hat{a}}_{ij}\right)+\left(1-a_{ij}\right)*\log\left(1-{\hat{a}}_{ij}\right)\right)$$ (9)

where $a_{ij}$ and ${\hat{a}}_{ij}$ are the elements of row *i* and column *j* of matrix *A* and row *i* and column *j* of reconstruction matrix $\hat{A}$ , respectively, and *N* is the number of nodes in the cell diagram. Since the number of cells in the cell diagram is *N*, the total number of elements in the matrix is N×N .

### Iterative process

The purpose of iteration is to build a better cell map and gradually converge through continuous iteration so that the cell map has more biological significance. The iterative process is defined as follows:

$$\tilde{A}=\mu L_{0}+\left(1-\mu \right)\frac{A_{ij}}{\underset{j}{\sum }A_{ij}}$$ (10)

where $L_{0}$ is the adjacency matrix of the pruned cell map and μ is the parameter controlling the iteration speed. $\tilde{A}$ is the symmetrically normalized adjacency matrix, and $A_{ij}$ represents elements of row i and column j of the adjacency matrix A. The iteration stops depending on two criteria: 1) whether the adjacency matrix converges and 2) whether the types of cells are as similar as possible as determined by similarity measurement. During the iteration, the parameter μ is set to 0.5. The clustering result obtained by the final iteration is the final clustering result.

### Imputation autoencoder

After the iteration stops, the imputation autoencoder imputes and denoises the raw gene expression matrix according to the relationship between cells and the cell types. The imputation autoencoder uses cell graph, type, and L1 regularizer (Wang et al., 2021). The regularizer of the cell graph is defined as:

$$\gamma_{1}\sum \left(A∙{\left(X-\hat{X}\right)}^{2}\right)$$ (11)

where A is the adjacency matrix obtained in the last iteration, and represents the product. The punishment of the edge of the cell graph in training is Eq. 11:

$$\gamma_{2}\sum \left(B∙{\left(X-\hat{X}\right)}^{2}\right)$$

$$B_{ij}=\left\{\begin{array}{l} 1\;i\;and\;j\;belong\;to\;the\;same\;cell\;type\\ 0\;else\; \end{array}\right.$$ (12)

where B is the relationship matrix of cells. When i and j are the same cell type, the value of $B_{ij}$ is 1, otherwise, the value is 0; $\gamma_{1}$ *,* $\gamma_{2}$ represent the intensity of regularization. The L1 regularizer is defined as:

$$\beta \sum \left|w\right|$$ (13)

where w is the weight, and $\sum \left|w\right|$ represents the sum of the absolute values of each element. By reducing the w , the generalization ability of the imputation autoencoder is improved and sparsity is increased. $\beta \epsilon \left[0,1\right]$ is the parameter that controls the intensity of the L1 regularizer. The loss function of the imputation autoencoder is defined as:

$$Loss=\left(1-\alpha \right)\sum {\left(X-\hat{X}\right)}^{2}+\alpha \sum \left({\left(X-\hat{X}\right)}^{2}*TRS\right)+\beta \sum \left|w\right|+\gamma_{1}\sum \left(A∙{\left(X-\hat{X}\right)}^{2}\right)+\gamma_{2}\sum \left(B∙{\left(X-\hat{X}\right)}^{2}\right)$$ (14)

### Evaluation metrics

To verify the imputation performance of the model, we flipped the random 10% non-zero data to zero. The gene imputation ability of the scGAEGAT model is verified by calculating the three evaluation indicators: median L1 distance, cosine similarity, and root mean squared error (RMSE). In Eqs 14–16, *x* represents the row vector of the original data and *y* represents the row vector after data imputation.

L1 distance represents the absolute deviation between the original and imputed data. The lower the value means, the higher the similarity, and the better the obtainable imputation effect. L1 distance is greater than zero:

$$L1\;distance=\left|x-y\right|$$ (15)

Cosine similarity refers to the product between the original and imputed data. The value range of cosine similarity is [0, 1]; the higher the value, the better the imputation performance.

$$Cosine\;similarity\left(x,y\right)=\frac{xy^{T}}{\left\|x\right\|\left\|y\right\|}$$ (16)

RMSE represents the square root of the quadratic mean of differences between the original data and the imputed data; the smaller the value, the better the effect.

$$RMSE\left(x,y\right)=\sqrt{\frac{\sum_{i=1}^{N}{\left(x_{i}-y_{i}\right)}^{2}}{N}}$$ (17)

The following evaluation indicators were used to estimate the performance of cell clustering: adjusted rand index (ARI), adjusted mutual information (AMI), normalized mutual information (NMI), completeness score, and silhouette coefficient score. The other cell clustering metrics have been published previously (Wang et al., 2021). We used the Louvain clustering method with the default parameters. The higher the values of the following metrics, the better the clustering performance.

The similarity between the current clustering results and the previously assigned cell pairs is expressed by calculating the ARI:

$$ARI=\frac{RI-E\left[RI\right]}{\max\left(RI\right)-E\left[RI\right]}$$ (18)

The unadjusted rand index (RI) is defined as:

$$RI=\frac{a+b}{C_{n}^{2}}$$ (19)

where *a* represents the number of correctly labeled cells in the same set, *b* represents the number of correctly labeled cells in different sets, and $C_{n}^{2}$ represents the total number of possible pairs.

AMI is similar to ARI and uses information entropy. A higher AMI indicates a higher similarity.

$$AMI\left(x,y\right)=\frac{MI\left(x,y\right)-E\left(MI\left(x,y\right)\right)}{Avg\left(H\left(x,y\right)\right)-E\left(MI\left(x,y\right)\right)}$$ (20)

where *x* and *y* represent the inferred and standard clustering results, respectively. *H* represents the number of uncertain clusters in a partition set, which is defined as follows:

$$H\left(x\right)=\overset{\left|x\right|}{\underset{i=1}{\sum }}P\left(i\right)\log\left(P\left(i\right)\right)$$ (21)

where $P\left(i\right)=\left|x_{i}\right|/N$ . *MI* is defined as:

$$MI\left(x,y\right)=\overset{\left|x\right|}{\underset{i=1}{\sum }}\overset{\left|y\right|}{\underset{j=1}{\sum }}P\left(i,j\right)\log\left(\frac{P\left(i,j\right)}{P\left(i\right)P\left(j\right)}\right)$$ (22)

NMI is another adjusted form of mutual information (MI). NMI is defined as:

$$NMI\left(x,y\right)=\frac{MI\left(x,y\right)}{mean\left(H\left(x\right),\;H\left(y\right)\right)}$$ (23)

where *H (x)* and *H (y)* are the entropy of *x* and *y*, respectively.

The completeness score (CS) measures how much all class members are assigned to the same cluster. Higher $CS\in \left[0,1\right]$ means a higher similarity.

$$Completeness=1-\frac{H\left(K/C\right)}{H\left(K\right)}$$ (24)

The silhouette coefficient score indicates how similar an object is to its cluster compared to others. Unlike ARI, it does not require the real label. The silhouette coefficient score is defined as:

$$Silhouette=\frac{b-a}{\max\left(a,b\right)}$$ (25)

where a represents the average distance between the object and other objects, and b represents the average distance between the object and all points in the nearest cluster.

## Results

To evaluate the dropout imputation and cell clustering performance of different methods for analyzing single-cell data sets, we compared scGAEGAT results with those of five other models, including single-cell variational inference (scVI) (Lopez et al., 2018), generative adversarial networks for scRNA-seq imputation (scIGANs) (Xu et al., 2020b), single-cell impute (scImpute) (Li and Li, 2018), deep count autoencoder network (DCA) (Eraslan et al., 2019), and DeepImpute (Arisdakessian et al., 2019). Each method was used to analyze data from four scRNA-seq data sets (Kolodziejczyk, Klein, Zeisel, and Chung) with gold standard cell labels (Klein et al., 2015; Kolodziejczyk et al., 2015; Zeisel et al., 2015; Chung et al., 2017). The scVI model is based on a hierarchical Bayesian model and uses deep neural networks to define conditional probabilities, which can accurately recover gene expression signals as well as impute zero-valued entries, potentially enhancing cell clustering without adding artifacts or false signals. The scIGANs generate data for a set of realistic single cells instead of directly borrowing information from observed cells to impute the dropout events, which helps avoid over-fitting for the abundant cell type in a population while maintaining enough imputation power for rare cells. DCA takes the count distribution, overdispersion, and sparsity of the data into account using a negative binomial noise model with or without zero inflation, and nonlinear gene-gene dependencies are captured. The scImpute model automatically identifies likely dropouts and only performs imputation of those values without introducing new biases into the data; it also detects outlier cells and excludes them from imputation. The DeepImpute model uses several sub-neural networks to impute groups of target genes using signals (genes) strongly associated with the target genes.

### Results of gene imputation

The synthetic dropout was adopted to simulate the imputation effects based on the same leave-one-out strategy used in scVI. The dropout rate was set at 10% and 30%, respectively, by randomly flipping a number of the non-zero entries to zeros. Cosine similarity, median L1 distance, and RMSE between the original data set and the imputed values for these synthetic entries were calculated to compare scGAEGAT dropout performance with that of scVI, scIGANS, scImpute, DCA, and DeepImpute.

**FIGURE 3:**
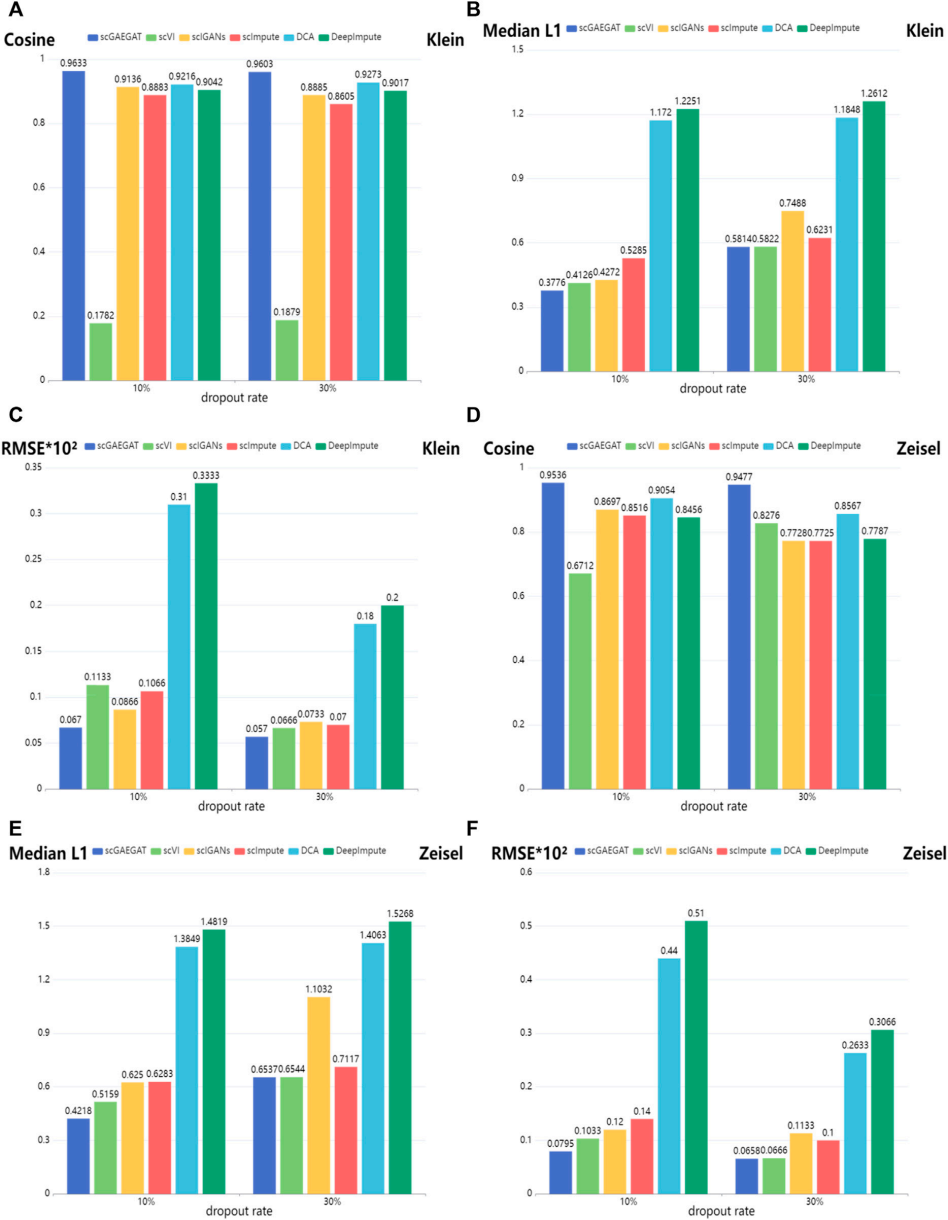
Gene imputation performance comparison of cosine similarity, median L1 distance, and RMSE scores between the scGAEGAT model and the five existing imputation methods, with settings of 10% and 30% dropout rate. **(A–C)** Results for Klein data sets. **(D–F)** Results for Zeisel data sets.

Figure 3 shows the results of cosine similarity (Cosine), median L1 distance (Median L1), and RMSE for Klein data sets (Figures 3A–C) and Zeisel data sets (Figures 3D–F) under dropout rates of 10% and 30%, respectively. Klein and Zeisel are large-scale data sets. The cosine similarity of scGAEGAT ranked highest with 10% and 30% dropout rates (Figure 3A), achieving values of 0.9633 and 0.9603, respectively. The results were better than those achieved using scVI (0.1782 and 0.1879), scIGANS (0.9136 and 0.8885), scImpute (0.8883 and 0.8605), DCA (0.9216 and 0.9273), and DeepImpute (0.9042 and 0.9017). The scVI had the worst imputation performance. The results of DCA were slightly worse than those generated by scGAEGAT. Figure 3B shows that the scGAEGAT model obtained the best results for median L1 distance (0.3776 and 0.5814), and the median L1 distance values generated using the DeepImpute model (1.2251 and 1.2612) were far greater than those from the scGAEGAT model, and therefore the worst results. According to Figure 3C, scGAEGAT demonstrated the best RMSE results (0.067 and 0.050) among the six methods tested. The results for the Zeisel and Klein data sets were similar. Figures 3D–F indicate that the scGAEGAT model had the best imputation performance. For large-scale data sets, scGAEGAT achieved the best results in recovering gene expression among the tested models, as demonstrated *via* cosine similarity, median L1 distance, and RMSE at the 10% and 30% synthetic dropout rates, respectively.

### Results of cell clustering

**FIGURE 4:**
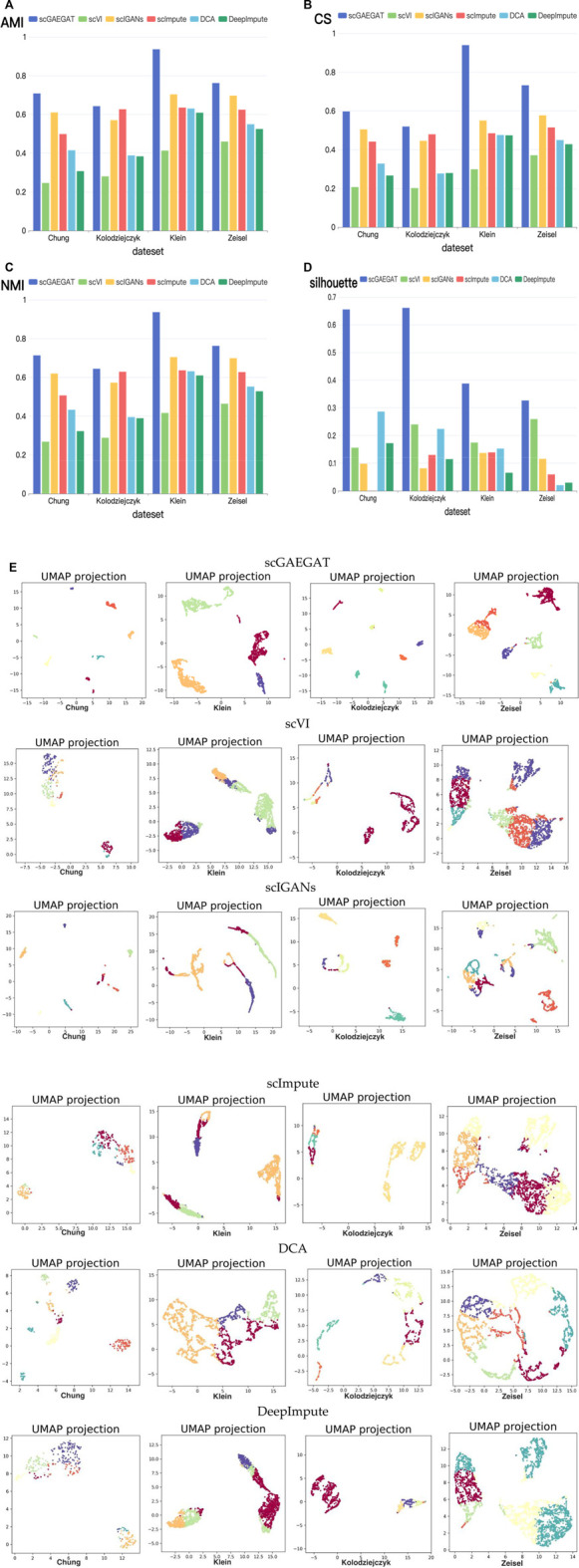
(Continued). Cell clustering performance comparison of adjusted mutual information **(A)**, completeness score **(B)**, normalized mutual information **(C)**, and silhouette coefficient score **(D)** between the scGAEGAT model and the five existing imputation methods using four data sets. **(E)** UMAP visualizations show the clustering effect of scGAEGAT compared with the other five models across four data sets.

The purpose of imputation is to improve the downstream analysis of scRNA-seq data. Therefore, we continued to evaluate the clustering performance of scGAEGAT to estimate the downstream analysis by the Louvain clustering algorithm. The results of cell clustering are illustrated in Figure 4. We used four metrics, including AMI, CS, NMI, and silhouette coefficient score (Silhouette), to compare scGAEGAT performance with five existing methods, including scVI, scIGANS, scImpute, DCA, and DeepImpute, using the Chung, Kolodziejczyk, Klein, and Zeisel data sets. The results are shown in Figures 4A–D. The greater the values of AMI, CS, NMI, and Silhouette, the better the model’s performance. We can see from Figures 4A–D that the scGAEGAT model had the best results. For the large-scale Klein data set, the completeness score was 0.9398. The cell clustering effect of scGAEGAT was significantly better than the other methods, which strongly supports the capability of scGAEGAT to capture real cell-cell communications and interactions. As shown in Figures 4A–C, the scVI model had the worst clustering performance, whereas the results from the scIGANS and scImpute models were better than those from scVI, DCA, and DeepImpute. Figure 4D demonstrates that, except for the scGAEGAT model, the clustering effect of the models tested was unsatisfactory. Using the scGAEGAT model, the Silhouette reached 0.65538 and 0.66105, respectively, in clustering small-scale data sets (Chung and Kolodziejczyk), which far exceeded the second- and third-ranking methods DCA and scVI (0.28653 and 0.24004, respectively).

Figure 4E demonstrates use of the UMAP method to more intuitively show the clustering effect of scGAEGAT, compared with the five other models, on four data sets. The results illustrate that the distance between cells and clusters within-group is the least using scGAEGAT, and the between-group distance is the most significant. When other models are used, cells are more separated between clusters, which intuitively shows that scGAEGAT has a strong capacity for capturing the relationships between cells.

### Analysis of the graph autoencoder mechanism of the scGAEGAT model

To address the significance of using the graph autoencoder in scGAEGAT, we performed the experiment with and without the GAE. Table 1 and Table 2 show the imputation results when the dropout rate was 10% and the head was 3 for the large-scale Klein data sets and small-scale Chung data sets, respectively. Table 3 and Table 4 show the results of clustering for the Klein and Chung data sets. Five evaluation metrics were used to estimate imputation performance: Mean L1 distance (L1Mean), Median L1 distance (L1Median), Max L1 distance (L1Max), cosine similarity (Cosine), and RMSE. Nine evaluation metrics were used to estimate the clustering performance of scGAEGAT: Silhouette, Davies Bouldin score (DBS), ARI, AMI, NMI, CS, Fowlkes mallows score (FMS), V measure score (VMS), and homogeneity score (HS). Bold numbers in all the tables indicate the best results. The data in Table 1 and Table 2 demonstrate that four values, the L1Mean, L1Median, L1Max, and RMSE, showed greater improvement with GAE than without. However, as indicated by the data in Table 3 and Table 4, the clustering effect of scGAEGAT with GAE was significantly better than that of scGAEGAT without GAE, in both large-scale and small-scale data sets. Although there was dramatic improvement of the model with GAE present (versus without GAE) according to nine evaluation metrics, the GAE is not fully involved in the imputation process in the scGAEGAT model because it is only used to reconstruct the cell graph and capture cell relationships.

**TABLE 1:** Imputation performance of scGAEGAT with and without GAE for Klein data sets.

	L1Mean	L1Median	L1Max	Cosine	RMSE*10^2^
With GAE	**0.4216**	**0.3776**	**4.8041**	**0.9633**	**0.0670**
Without GAE	0.4244	0.3797	4.9719	**0.9633**	0.0674

The bold values are the best results.

**TABLE 2:** Imputation performance of scGAEGAT with and without GAE for Chung data sets.

	L1Mean	L1Median	L1Max	Cosine	RMSE*10^2^
With GAE	**1.6959**	**1.4889**	**9.6577**	0.9294	**0.7785**
Without GAE	1.7250	1.5458	**9.6577**	**0.9301**	0.7919

The bold values are the best results.

**TABLE 3:** Clustering performance of scGAEGAT with and without GAE for Klein data sets.

	Silhouette	DBS	ARI	AMI	NMI	CS	FMS	VMS	HS
With GAE	**0.3878**	**1.2023**	**0.9601**	**0.9264**	**0.9365**	**0.9398**	**0.9713**	**0.9365**	**0.9332**
Without GAE	0.2129	1.7219	0.6463	0.6899	0.6902	0.6815	0.7430	0.6902	0.6993

The bold values are the best results.

**TABLE 4:** Clustering performance of scGAEGAT with and without GAE for Chung data sets.

	Silhouette	DBS	ARI	AMI	NMI	CS	FMS	VMS	HS
With GAE	**0.6553**	**0.7125**	**0.5534**	**0.7082**	**0.7137**	**0.5973**	**0.6805**	**0.7137**	**0.8866**
Without GAE	0.1736	1.8295	0.5457	0.6873	0.6932	0.5794	0.6744	0.6932	0.8626

The bold values are the best results.

### Analysis of the graph attention mechanism of the scGAEGAT model

**FIGURE 5:**
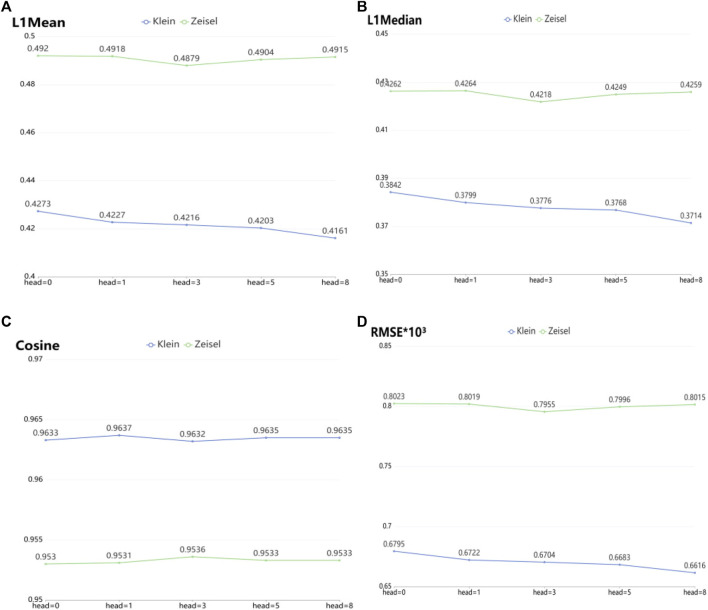
Performance of imputation of scGAEGAT without graph attention (head = 0) and with graph attention (head = 1, head = 3, head = 5, head = 8) according to evaluation metrics L1Mean **(A)**, L1Median **(B)**, cosine similarity **(C)**, and RMSE **(D)**.

To verify the imputation performance using GAT in scGAEGAT, we performed the experiment with and without GAT. As shown in Figure 5, we evaluated the performance of five models on the large-scale Klein and Zeisel data sets. They are scGAEGAT without GAT (head = 0), with 1 attention head, 3 attention heads, 5 attention heads, and 8 attention heads. According to L1Mean (Figure 5A), L1Median (Figure 5B), Cosine (Figure 5C), and RMSE (Figure 5D) data (smaller values indicate better imputation performance), use of the scGAEGAT model with GAT produces better results than without GAT. When the attention head is 8, the effect of imputation is best for the Klein data set. For the Zeisel data set, imputation has a comparative advantage when the attention head is 3. We also found that the performance of scGAEGAT with the single-head attention mechanism (1 attention head) was not stable, which suggests that use of a multi-head attention mechanism with the scGAEGAT model will provide the best results.

**FIGURE 6:**
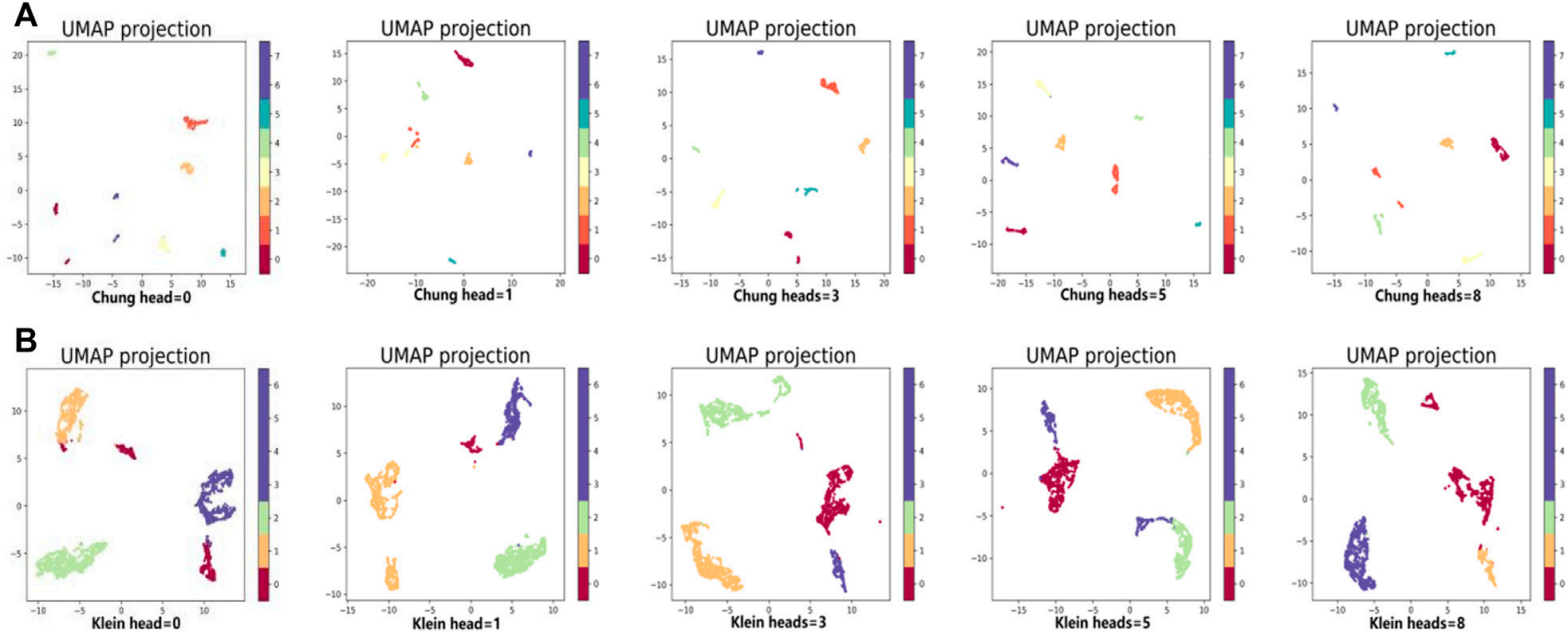
Performance of cell clustering of scGAEGAT without graph attention (head = 0) and with graph attention (head = 1, head = 3, head = 5, head = 8). **(A)** Chung data sets. **(B)** Klein data sets.

In the same way, we used the above five models to evaluate the effect of GAT on cell clustering. Table 5 and Table 6 display Silhouette, DBS, ARI, AMI, NMI, CS, FMS, VMS, and HS results for the small-scale Chung and large-scale Klein data sets. Figure 6 displays the results of clustering by UMAP. Taken together, the data indicate that the performance of the scGAEGAT model using the attention mechanism is superior to use of the model without attention. Overall, when the head is 3, clustering performance is optimal.

**TABLE 5:** Clustering performance of scGAEGAT with and without GAT for Chung data sets.

	Silhouette	DBS	ARI	AMI	NMI	CS	FMS	VMS	HS
Without GAT	0.6574	0.6706	0.5250	0.6670	0.6733	0.5616	0.6577	0.6733	0.8406
1 attention head	0.5950	0.7318	0.5495	0.7005	0.7062	0.5910	0.6772	0.7062	0.8770
3 attention heads	0.6553	0.7125	**0.5534**	**0.7082**	**0.7137**	**0.5973**	**0.6805**	**0.7137**	**0.8866**
5 attention heads	0.6534	0.7088	0.5468	0.6944	0.7003	0.5859	0.6751	0.7003	0.8701
8 attention heads	**0.6735**	**0.6479**	0.5530	0.7024	0.7080	0.5922	0.6803	0.7080	0.8801

The bold values are the best results.

**TABLE 6:** Clustering performance of scGAEGAT with and without GAT for Klein data sets.

	Silhouette	DBS	ARI	AMI	NMI	CS	FMS	VMS	HS
Without GAT	0.3733	1.5436	0.8352	0.8187	0.8189	0.8078	0.8803	0.8189	0.8303
1 attention head	0.3447	1.2099	0.7826	0.8275	0.8278	0.8514	0.8470	0.8278	0.8054
3 attention heads	0.3878	1.2023	**0.9601**	**0.9364**	**0.9365**	**0.9398**	**0.9713**	**0.9365**	**0.9332**
5 attention heads	0.3866	1.4118	0.8361	0.8255	0.8257	0.8133	0.8809	0.8257	0.8386
8 attention heads	**0.4043**	**1.1677**	0.8143	0.8209	0.8211	0.8263	0.8669	0.8211	0.8160

The bold values are the best results.

## Discussion

Today, researchers are still exploring dimensional reduction, gene imputation (dropout events), and cell clustering of scRNA-seq data. However, solving issues related to the heterogeneity of single cells is still a difficult challenge. This study demonstrated the development and testing of scGAEGAT, a model based on graph neural networks, in cell clustering and imputation of data to represent missing genes. Multi-modal GAEs and GAT mechanisms were added to the basic graph convolution neural (GCN) network to improve the accuracy of single-cell data analysis. Our results show that the scGAEGAT model delivered the best performance according to various evaluation metrics. Moreover, the scGAEGAT model showed better results than any of the existing models in analysis of both large-scale and small-scale data sets.

GAE is an unsupervised learning framework, which aims to learn low-dimensional node vectors through an encoder, and then reconstruct graph data through a decoder. A GAT network is similar to GCN and seeks aggregation function to fuse adjacent nodes in the graph. The attention mechanism has almost become a standard configuration in sequential tasks. Its value lies in focusing on the essential part of the object. The contributions of the attention mechanism to the graph neural network are three-fold: assigning attention weights to different neighbors when aggregating feature information, integrating multiple models according to attention weights, and using attention weight to guide random walk. Therefore, the GAT mechanism of the scGAEGAT model can add different weights to different cells and focus on the most relevant parts, which enhances the likelihood of discovering potential node relationships. With GAEs, the scGAEGAT model has improved capacity to discover relationships between similar cells in aggregate. Compared with other imputation methods, scGAEGAT demonstrated advantages in data sets of different sizes (especially in large data sets), suggesting scGAEGAT is a generalizable method with potentially widespread applicability.

In future studies, we will investigate scRNA-seq data analyses based on deep learning to identify an optimal framework. This deep learning framework is also significant in its potential use for data analysis in other omics specialties to promote the development of bioinformatics.

## Data Availability

The original contributions presented in the study are included in the article/Supplementary Material; further inquiries can be directed to the corresponding authors.
